# Heart rate variability as predictor of mortality in sepsis: A systematic review

**DOI:** 10.1371/journal.pone.0203487

**Published:** 2018-09-11

**Authors:** Fábio M. de Castilho, Antonio Luiz P. Ribeiro, Vandack Nobre, Guilherme Barros, Marcos R. de Sousa

**Affiliations:** 1 Hospital das Clínicas and School of Medicine, Universidade Federal de Minas Gerais (UFMG), Belo Horizonte, Brazil; 2 Núcleo Interdisciplinar de Investigação em Medicina Intensiva (NIIMI), UFMG, Belo Horizonte, Brazil; University of Palermo, ITALY

## Abstract

**Background:**

Autonomic dysregulation is one of the recognized pathophysiological mechanisms in sepsis, generating the hypothesis that heart rate variability (HRV) can be used to predict mortality in sepsis.

**Methods:**

This was a systematic review of studies evaluating HRV as a predictor of death in patients with sepsis. The search was performed by independent researchers in PubMed, LILACS and Cochrane, including papers in English, Portuguese or Spanish, indexed until August 20^th^, 2017 with at least 10 patients. Study quality was assessed by Newcastle-Ottawa Scale. To analyze the results, we divided the articles between those who measured HRV for short-term recordings (≤ 1 hour), and those who did long-term recordings (≥ 24 hours).

**Results:**

Nine studies were included with a total of 536 patients. All of them were observational studies. Studies quality varied from 4 to 7 stars in Newcastle-Ottawa Scale. The mortality rate in the studies ranged from 8 to 61%. Seven studies performed HRV analysis in short-term recordings. With the exception of one study that did not explain which group had the lowest results, all other studies showed reduction of several HRV parameters in the non-survivors in relation to the surviving septic patients. SDNN (Standard deviation of the Normal to Normal interval), TP (Total Power), VLF (Very Low Frequency Power), LF (Low Frequency Power), LF/HF (Low Frequency Power / High Frequency Power), nLF (Normalized Low Frequency Power), α1/α2 (short-term and long-term fractal scaling coefficients from DFA) and r-MSSD (Square root of the squared mean of the difference of successive NN-intervals) of the non-survivor group were reduced in relation to the survivors in at least one study. Two studies found that SDNN is associated with mortality in sepsis, even after adjusting for possible confounding factors. Three studies performed HRV analysis using long-term recordings. Only one of these studies found difference between surviving and non-surviving groups, and even so, in only one HRV parameter: LogHF.

**Conclusions:**

Several HRV parameters are reduced in nonsurviving septic patients in short-term recording. Two studies have found that SDNN is associated with mortality in sepsis, even after adjusting for possible confounding factors.

## Introduction

Sepsis, a syndrome in which there is dysregulated host response to infection and presence of organ dysfunction[1], has a high mortality rate that can vary between 10 and 50%[1, 2]. In addition to high lethality, its incidence has increased significantly in recent decades, making sepsis a serious global health problem [3]. For all these reasons, it is useful to identify the most serious septic patients through predictive scoring systems. Although autonomic dysregulation is one of the recognized pathophysiological mechanisms in sepsis[4], existing predictive scoring systems such as APACHE II (Acute Physiology and Chronic Health disease Classification System II)[5], SOFA (Sepsis-related Organ Failure Assessment)[6], SAPS-3 (Simplified Acute Physiology Score III)[7, 8] and MODS (Multiple Organ Dysfunction *Score)[9]* do not considers in their composition changes in the autonomic nervous modulation.

Physiological variation of heart rate indicates heart's capacity to adapt to different situations, and is influenced, among other factors, by the autonomic nervous system [10]. Heart rate variability (HRV) measures oscillations of the intervals between consecutive heart beats, being therefore a noninvasive indirect test to evaluate autonomic function [11]. Studies have shown that patients with sepsis have reduced HRV compared to healthy patients[12, 13]. Ahmad et al. demonstrated, in a small study, that patients with sepsis showed a significant drop in the value of several HRV parameters on average 35 hours before the diagnosis of sepsis[14]. These findings raised the possibility that the HRV can be used to predict the risk of developing sepsis or even for the diagnosis of sepsis. In addition to the diagnosis of sepsis, HRV parameter reduction seems to be related to worse outcomes in septic patients, and has a correlation with APACHE II and SOFA[15]. Finally, some studies have shown that HRV can be used to predict the risk of septic patients develop septic shock [16] and multiple organ dysfunction[17]. Recently, our research group published the results of a cohort study with septic patients in which several parameters of HRV were reduced in those patients who died in comparison to their counterparts. [18].

The objective of this study was to perform a systematic review of studies evaluating HRV as a predictor of death in patients with sepsis.

## Materials and methods

Following the PRISMA statement [19] for systematic reviews and specific guidelines for nonrandomized studies [20], three bibliographic methods were used to identify potential abstracts or investigations: remote search in electronic databases; evaluation of bibliographic citations from hand search of texts; and email contact with authors (see S1 File for PRISMA Checklist and S2 File for PRISMA Flow Diagram). The databases used were PubMed, LILACS and Cochrane. Independent reviewers participated in the search and selection of studies. Two independent reviewers (FMC and GB) made the search and selection of studies in the databases, while MRS resolved any divergences. Additional articles were searched by citation tracking of review articles and original articles, and by looking for additional articles authored by the same authors of the papers previously selected. After *analyzing titles and abstracts*, the selected articles were read in full to confirm eligibility, and doubts or disagreements were solved through discussions with senior researchers (ALPR, VN and MRS). Inclusion criteria were clearly defined before the beginning of search. This systematic review has been registered within PROSPERO (the NIHR International Prospective Register of Systematic Reviews), under the registration number CRD42017062367.

We included studies containing more than 10 patients which evaluated heart rate variability as predictor of mortality in sepsis, published before August 20^th^, 2017. Review studies and case series were excluded from this review. Publication languages included English, Portuguese and Spanish. The search-terms used were: "("Systemic Inflammatory Response Syndrome"[Mesh] OR "Systemic Inflammatory Response Syndrome" [All Fields] OR "Sepsis"[Mesh] OR "sepsis"[All Fields]) AND (("heart rate"[MeSH Terms] OR heart rate[Text Word]) AND (variability[Text Word] OR turbulence[All Fields]) OR "Nonlinear Dynamics"[Mesh] OR "Entropy"[Mesh] OR “triangular index”) AND (incidence[MeSH] OR mortality[MeSH] OR follow-up studies[MeSH] OR prognos*[Text Word] OR predict*[Text Word] OR course*[Text Word])”. Besides textual and MeSH terms selection, hand search within each paper’s references, and also "related citations", a search tool available in PubMed, were used to increase sensitivity of the search.

Two researchers (MRS and FMC) independently double checked the extraction of primary data from each study. Discrepancies were solved by consensus after discussion with the remaining researchers. The following information was extracted: study design and methodological data; demographic and clinical characteristics of patients; number of patients who died and mean or median values of each HRV parameter in the surviving and non-surviving groups.

The Newcastle-Ottawa Scale[21] was used to assess the quality of the included studies. Using this 'star system' (ranges from 0 to 9) each included study was judged on three broad perspectives, as recommended by the Cochrane Non-Randomized Studies Methods Working Group Version 5.1.0 [20, 22]: the selection of the study groups; the comparability of the groups; and the ascertainment of outcome of interest.

To analyze the results, we divided the articles between those who measured HRV for short-term recordings (≤ 1 hour), and those who did long-term recordings (≥ 24 hours), since we know that long-term recording have different oscillatory components as compared to those of short duration[11].

One of the included studies contained the median value of SDNN for surviving and non-surviving groups, but did not report the p-value of the comparison between groups. So, we estimated the mean and standard deviation of SDNN of each group on basis of the sample’s reported median and range according to the method devised by S.P. Hozo, B. Djulbegovic, and I. Hozo[23]. Subsequently, SDNN of survivors and nonsurvivors were compared using the Student´s *t* test, conducted in SPSS version 23 (SPSS Inc., Chicago, IL, USA).

## Results

**Fig 1:**
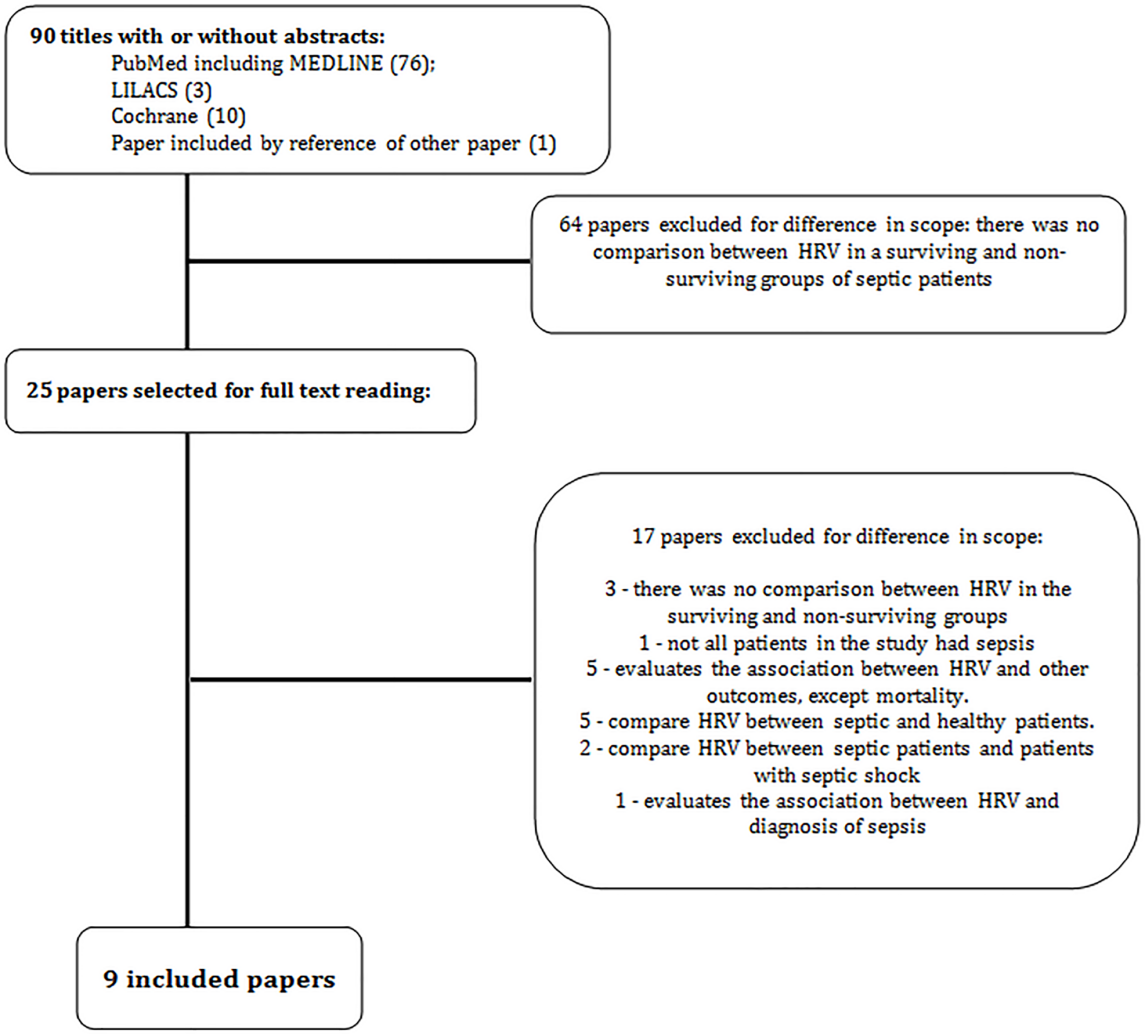
Inclusion flow of studies.

The selection process and the inclusion flow of studies are shown in Fig 1. Nine studies were included, with a total of 536 patients [18, 24–31]. Table 1 shows the main methodological characteristics of the studies, and Table 2 reported the rate for each item of the Newcastle-Ottawa Scale, while Table 3 describes the methodologies used in each study to measure HRV.

**Table 1:** Characteristics of included studies.

Study (1st author/year)	Country	Enrollment period	Sample Size	Age (Mean)	Male (%)	Mortality Endpoint	Mortality Rate (%)	Definition of sepsis	Population (Septic patients)	Exclusion criteria
Tateishi 2007	China	2002 to 2005	45	54	71	?	29	Infectious SIRS	Adults in the ICU	DM or neurological disease
Nogueira 2008	Brasil	2003 to 2005	31	51	74	In-hospital mortality	61	Infectious SIRS	Adults in the ICU receiving mechanical ventilation	MI, nonsinusal rhythm, use of a permanent pacemaker, CHF class III or IV, or DM
Chen 2008	Taiwan	2006	132	67	47	In-hospital mortality	8	Infectious SIRS	Adults in the ED	Arrhythmia, cardiac pacing or respiratory failure under mechanical ventilator
Papaioannou 2009	Greece	2007 to 2008	20	58	76	?	20	?	Adults in the ICU receiving mechanical ventilation	Atrial flutter or fibrillation, ventricular ectopic beats, use of anti-arrhythmic medication, severe brain injuries or acquired immunodeficiencies
Duque 2012	Colombia	2009 to 2010	100	55	58	?	40	?	Adults in the ICU with the need for cardiovascular or ventilatory support	Clinical or electrocardioggraphic features complicating interpretation of the Holter recordings or coronary disease
Chen 2012	Taiwan	?	64	?	?	24-hour mortality	25	Infectious SIRS	Age- and sex-matched patients with sepsis in the ED used as the negative controls	Persistent arrhythmia or cardiac pacing
Brown 2013	USA	2009 to 2011	48	57	46	28-day mortality	10	Infectious SIRS	>15 years of age patients in the ICU with severe sepsis or septic shock	Pregnancy or non-sinus rhythm
Cedillo 2015	Spain	2012	33	62	39	In-hospital mortality	18	Infectious SIRS	Non-smoking patients admitted to the ward	Malignant diseases, CHF, nonsinusal rhythm, COPD, immunosuppression, use of beta or calcium-channel blockers, poorly-controlled DM, liver or renal failure or age > 80 years.
Castilho 2017	Brasil	2012 to 2014	63	53	60	28-day mortality	25	Infectious SIRS	Adults in the ICU	Antibiotic therapy for more than 48 hours prior to enrollment, nonsinus rhythm or with pacemaker

ICU = Intensive Care Unit; SIRS = Systemic Inflammatory Response Syndrome; ED = Emergency Department; DM = diabetes mellitus; MI = myocardial infarction; CHF = chronic heart failure; COPD = chronic obstructive pulmonary disease

**Table 2:** Newcastle-Ottawa Scale.

Study (1st author/year)	Selection 1	Selection 2	Selection 3	Selection 4	Comparability	Outcome 1	Outcome 2	Outcome 3	Total Score
Tateishi 2007	0	1	1	1	0	1	0	1	5
Nogueira 2008	0	1	1	1	0	1	1	1	6
Chen 2008	1	1	1	1	0	1	1	1	7
Papaioannou 2009	1	1	0	1	0	1	0	0	4
Duque 2012	1	1	1	1	0	1	1	0	6
Chen 2012	0	1	1	1	0	1	0	1	5
Brown 2013	1	1	1	1	0	1	1	1	7
Cedillo 2015	0	1	0	1	0	1	1	0	4
Castilho 2017	1	1	1	1	0	1	1	1	7

**Table 3:** HRV measurement in included studies.

Study (1st author/year)	Duration of measurement	Recording period used for analysis	Equipment	Recording day	Patient´s conditions during record	Were there patients receiving Mechanical Ventilation?	HRV parameters
Tateishi 2007	24-hour	24-hour	Monitor	First and last	?	Yes	Frequency domain: LF, HF
Nogueira 2008	30-minute	?	Holter	1,3 and 6	Supine position, with ventilatory parameters completely controlled by the ventilator	All	Frequency domain: LF, HF, LF/HF
Chen 2008	10-minute	The last 512 R-R intervals	ECG	First	Supine position, room temperature around 25°C	No	Time domain: SDNN, r-MSSD; Frequency domain: TP, VLF, LF, HF, nVLF, nLF, nHF and LF/HF
Papaioannou 2009	10-minute	128 seconds time serie	Holter	?	Supine position	All	Time domain: SDNN; Frequency domain: LF, HF, LF/HF; Nonlinear method: SD1/SD2
Duque 2012	48-hour	?	Holter	First	?	Yes	Time domain: SDNN, pNN50
Chen 2012	10-minute	The last 512 R-R intervals	ECG	First	?	Yes	Time domain: SDNN, CV; Frequency domain: TP, VLF, LF, HF, LF/HF
Brown 2013	6-hour	First 30minutes	Monitor	First	?	Yes	Time domain: NN, SDNN, r-MSSD, pNN50, NN50; Frequency domain: TP, LF, HF, LF/HF; Nonlinear methods: SD1/SD2, Sample entropy, DFA short-term coefficient, DFA long-term coefficient, Ratio of DFA coefficients
Cedillo 2015	15-minute	The last 512 R-R intervals	ECG	First	Supine position after a 10-min resting period and normal breathing	No	Time domain: r-MSSD; Frequency domain: TP, LF, HF, LF/HF
Castilho 2017	20-minute and 24-hour	First 10 minutes	Holter	First	Supine position and no intervention was made during its recording	Yes	Time domain: NN, SDNN, r-MSSD, pNN50; Frequency domain: TP, VLF, LF, HF, LF/HF

LF = Low Frequency Power; HF = High Frequency Power; LF/HF = Low Frequency Power / High Frequency Power; TP = Total Power; VLF = Very Low Frequency Power; nVLF = Normalized Very Low Frequency; nLF = Normalized Low Frequency Power; nHF = Normalized High Frequency Power; NN = Normal-to-Normal average interval; SDNN = Standard deviation of the NN interval; r-MSSD = Square root of the squared mean of the difference of successive NN-intervals; pNN50 = Percentage of NN intervals deviated by more than 50 ms from adjacent NN-intervals; NN50 = Number of pairs of adjacent NN intervals differing by more than 50 ms in the entire recording; SD1 = Poincare standard deviation 1; SD2 = Poincare standard deviation 2; DFA = Detrended Fluctuation Analysis; CV = Coefficient of variation

Study quality analysis by the Newcastle-Ottawa Scale showed that, in general, studies were representative of the sampled population, varying from 4 to 7 stars (mean 5.7). Except for the study of Chen and cols. 2012[27], in which patients with sepsis were included as controls of successfully resuscitated patients with out-of-hospital cardiac arrest (the main population of interest in the study), all other studies were prospective cohorts primary designed to include/primary focused on septic patients. One of the studies[25] was a combined prospective cohort and case-control study. The cohort group consisted of 33 septic patients, and these were the data we used in this review. Regarding the diagnostic criteria for sepsis used in each study, in two studies[28, 30] this information is not clearly reported, while in the other 7 studies[18, 24–27, 29, 31] the presence of infection and SIRS was used as the diagnostic criteria. Of these seven studies, two[24, 31] use the 1992 Consensus[32], while five[18, 25–27, 29] use the 2001 consensus[33].

Taken as whole, the included studies measured the following HRV parameters in the time domain: Normal-to-Normal (NN) average interval, Standard deviation of the NN interval (SDNN), Square root of the squared mean of the difference of successive NN-intervals (r-MSSD), Percentage of NN intervals deviated by more than 50 ms from adjacent NN-intervals (pNN50), Number of pairs of adjacent NN intervals differing by more than 50 ms in the entire recording (NN50), Coefficient of variation (CV); frequency domain: Low Frequency Power (LF), High Frequency Power (HF), ratio of LF to HF (LF/HF), Total Power (TP), Very Low Frequency Power (VLF), Normalized Very Low Frequency (nVLF), Normalized Low Frequency Power (nLF) and Normalized High Frequency Power (nLF); nonlinear methods: Poincare standard deviation 1 (SD1), Poincare standard deviation 2 (SD2), Short-term (α1) and long-term (α2) fractal scaling coefficients from Detrended Fluctuation Analysis (DFA).

Of the nine studies included in this systematic review, five make explicit the realization of some type of treatment for artifacts (period selection without artifact, manual deletion of artifacts, deletion of record if it presents artifact percentage greater than a predetermined value etc.). The way this was done in each study can be seen in the S1 Table.

The small number of studies, their technical limitations and its great heterogeneity prevented a meta-analysis to be performed. Some studies only presented HRV results in graphs [29, 31] or summary descriptions in the body of the article[24, 25, 30], without informing the central value of each HRV parameter in the surviving and non-surviving groups. The outcome of one study was 24-hour mortality[27], not allowing comparison with the other studies, which assessed mortality at longer time interval: 28-day mortality in two studies[18, 24], the in-hospital mortality in three studies[25, 26, 29] and, although the time at which endpoint mortality is assessed is unclear in the remaining three studies[28, 30, 31], the presented data strongly suggest that the follow-up for this outcome was longer than 24h, probably being in-ICU mortality. There was also a large difference in the population evaluated in the included studies: for instance, while two of the studies excluded mechanically ventilated patients[25, 26], two other studies restricted their analysis to patients who were on mechanical ventilation[29, 30]. The mortality rate observed in the different studies varied considerably, ranging from 8 to 61%, probably indicating a difference in clinical spectrum among populations.

Seven studies performed HRV analysis in short-term recordings (≤ 1 hour) [18, 24–27, 29, 30], Three studies performed HRV analysis using long-term recordings (≥ 24 hours) [18, 28, 31]. One of the articles [24] did an intermediate period recording (6 hours), but in this paper HRV analysis was restricted to the first 30 minutes of recording. Therefore, this study was included in the short-term recording (<1 hour) group. We did not find any article that did HRV analysis for period between 1 hour and 24 hours. The main results of these studies are presented in Table 4 and Table 5, respectively. One of the studies made both a recording of short duration (20 minutes) and another one of long duration (24 hours)[18].

**Table 4:** Main results in short time record studies.

Study (1st author/year)	Duration of measurement	Does it show the values (mean or median) of HRV parameters in the surviving and non-survivors groups?	How are the results presented?	HRV parameters of nonsurvivors lower than those of survivors*	HRV parameters of nonsurvivors higher than those of survivors*	Additional results / Comments
Nogueira 2008	30-minute	No	Graph comparing LF, HF e LF/HF between surviving and non-surviving patients.	LF, HF and LF/HF		
Chen 2008	10-minute	Yes	Table with HRV parameters values for surviving and non-surviving groups	SDNN, TP, VLF, LF and LF/HF	nHF	Multiple logistic regression model identified SDNN and nHF as the significant independent variables in the prediction mortality.
Papaioannou 2009	10-minute	No	Only citation in the text of the article			The natural logarithms of SDNN, LF and HF were significantly different between survivors and non-survivors, but there is no information on which patient group had the highest values.
Chen 2012	10-minute	Yes	Table with HRV parameters values for surviving and non-surviving groups	nLF, and LF/HF	nHF and HF	
Brown 2013	30-minute	No	Only citation in the text of the article	α1/α2		
Cedillo 2015	15-minute	No	Only citation in the text of the article	r-MSSD and nHF		
Castilho 2017	20-minute	Yes	Table with HRV parameters values for surviving and non-surviving groups	SDNN, TP, VLF, LF and LF/HF		SDNN ≤17 is a risk factor for death in septic patients, even after adjusting for APACHE II or SOFA.

* = Only results with statistical significance were shown

LF = Low Frequency Power; HF = High Frequency Power; LF/HF = Low Frequency Power / High Frequency Power; TP = Total Power; VLF = Very Low Frequency Power; nLF = Normalized Low Frequency Power; nHF = Normalized High Frequency Power; NN = Normal-to-Normal average interval; SDNN = Standard deviation of the NN interval; r-MSSD = Square root of the squared mean of the difference of successive NN-intervals; DFA = Detrended Fluctuation Analysis; CV = Coefficient of variation; α1/α2 = short-term and long-term fractal scaling coefficients from DFA

**Table 5:** Main results in long time record studies.

Study (1st author/year)	Duration of measurement	Does it show the values of HRV parameters in the surviving and non-survivors groups?	How are the results presented?	HRV parameters of nonsurvivors lower than those of survivors*	HRV parameters of nonsurvivors higher than those of survivors*	Additional results / Comments
Tateishi 2007	24-hour	No	Graph comparing logLF and logHF between surviving and non-surviving patients		LogHF	
Duque 2012	48-hour	Yes	Table with SDNN and PNN50 values for surviving and non-surviving groups			Median SDNN non significantly higher in the surviving group than in the nonsurvivor group (72.5ms [IQR 42] vs 61ms [IQR 65],p value not reported in the article, but we calculated p = 0.272)
Castilho 2017	24-hour	Yes	Table with HRV parameters values for surviving and non-surviving groups			There was no statistically significant difference in any HRV parameter measured in the 24 hours Holter between the two subgroups

* = Only results with statistical significance were shown

Log = logarithm; LF = Low Frequency Power; HF = High Frequency Power; NN = Normal-to-Normal average interval; SDNN = Standard deviation of the NN interval

Regarding the studies that used short term recordings to measure HRV, one of them reported a statistically significant difference of SDNN, LF and HF values between survivors and non-survivors, but authors did not inform which patient group had the highest values, what does not allow a deeper analysis of these results and comparison with other studies[30]. The remaining studies (n = 6) showed reduction of several HRV parameters in the non-survivors in relation to the surviving septic patients: SDNN[18, 26], TP[18, 26], VLF[18, 26], LF[18, 26, 29], LF/HF ratio[18, 26, 27, 29], nLF[27], α1/α2[24] and r-MSSD[25]. From these studies, four did not show the exact central value (mean or median) of the HRV parameters in survivor and non-survivor groups, presenting the results only in graphics or summarized in the body of the article. Chen et al, through the multiple logistic regression model, found that SDNN was a significant independent variable in the prediction of mortality in sepsis, with odds ratio of 0.719 (0.537–0.962), p = 0.026 [26]. Castilho et al defined a cut-off point for the SDNN of 17ms and found that Cox regression for dichotomous SDNN adjusted by the APACHE II showed Hazard ratio (HR) of 5.5 (1.2±24.8; p = 0.027) and Cox regression for this dichotomous variable adjusted by the SOFA showed HR of 6.3 (1.4±28.0; p = 0.015) [18].

There was a contradiction in the outcome prediction of HF and nHF, where some studies showed that their values were reduced in the non-survivor group [25, 29], while other studies showed higher values of these parameters were in the same group [26, 27].

From the studies using long-term recordings, only one article found statistically significant differences of any HRV parameter between survivors and non-survivors: LogHF was higher in the non-survivor than the survivor group[31]. The other two articles found no statistically significant differences between survivors and non-survivors for any HRV parameter[18, 28].

## Discussion

In this systematic review, we found that HRV parameters measured in short-term recordings were reduced in septic patients who died in relation to those who survived. This finding raises the possibility that HRV measurement can be a useful tool to predict the risk death in sepsis. On the other hand, there was no clear evidence of association between HVR parameters in long-term recordings and sepsis outcome.

There have has been an increasing interest in the role played by the autonomic nervous system in the complexes mechanisms involved in sepsis physiopathology. It is known, for example, that vagus nerve stimulation increases the secretion of corticotropin-releasing hormone, ACTH and cortisol[34]; it has been demonstrated that vagotomy attenuates fever response[35]; and that acetylcholine, the main vagal neurotransmitter, has an anti-inflammatory effect, attenuating the release of cytokines such as TNF, IL-1beta, IL-6 and IL-18, and preventing the development of shock[36]. Taken together, these findings suggest that the autonomic nervous system is involved in peripheral cytokine-to-brain communication, participating in the pathophysiology of sepsis.

Based on these results, some authors have investigated if the measurement of HRV, a noninvasive indirect test to evaluate autonomic function, in septic patients could be useful to predict outcome in these patients. HRV is measured using simple and non-invasive methods, requiring automated devices available on the market. Therefore, HRV is considered one of the most popular methods used to evaluate the autonomic function, being suitable for use in emergency department, ward or intensive care settings, where septic patients are usually taken[11].

The seven studies that analyzed HRV in short-term recordings[18, 24–27, 29, 30] showed significant difference between groups of surviving and non-surviving septic patients regarding different parameters. Except for one study that did not report which group had the highest value[30], the other six studies showed a reduction of at least one HRV parameter in the group of patients who died. These findings suggest that loss of heart rate oscillatory capacity, controlled, among other factors, by the autonomic nervous system is related to the severity of sepsis and risk of death. The only conflicting results revealed by this systematic review referred to the HF Power, which was shown to be reduced in the non-survivor group in some studies, but increased in others. HF Power reflects the vagal activity (parasympathetic) on the sinus node[37]. Different factors could explain the conflicting results for HF Power, such as the fact that the studies have small samples, the heterogeneity of the populations (some only with patients on mechanical ventilation, others only with patients on spontaneous ventilation, for example) or the presence of artifacts in electrocardiographic records. Of the nine studies included in this systematic review, five make explicit the realization of some type of treatment for artifacts. Studies show that the presence of artifacts or different treatment given to them may inflate the HRV analysis result[38, 39].

Among all HRV parameters tested to predict risk of death in sepsis, several parameters in both time domain and frequency domain have been shown to be reduced in non-surviving septic patients. More studies are needed to define which HRV parameters are most useful to predict mortality in sepsis and which cut-off values of each parameter should be used. [18, 26, 27, 29][18, 26]However, the SDNN stands out in the studies carried out until now, because two studies found this parameter as being associated with sepsis mortality, even after adjusting for possible confounding factors[18, 26] and one of these studies have even tested a cut-off point for this parameter[18]. SDNN seems to reflect all the cyclic components responsible for HRV (including sympathetic and parasympathetic activity)[11]. Some studies used the ICU monitors themselves to perform the electrocardiographic recording[24, 31], assuming that, through the implementation of SDNN calculation software, the ICU monitors themselves could calculate the SDNN of septic patients as a measure of the risk of death.

Only one [31] from the three [18, 28, 31] studies that analyzed long-term recordings for HRV found differences between surviving and non-surviving groups, and even so, in only one HRV parameter. We believe that the difficulty of association between HRV parameters and mortality in sepsis in long-term recordings is due, among other factors, to the dynamic condition of sepsis, in which metabolic disturbances, hemodynamic and ventilatory evolutionmay interfere with HRV parameters. In short-term recordings, it is possible to keep the patient in a specific position (supine, for example) and keep the patient without interventions such as orotracheal aspiration, which could interfere with HRV. Furthermore, in critical care patients, shorter periods of recording minimizes the interference with ICU routine activities, and have the advantage of being a fast tool for definition of severity, as these patients present immediate risk of death.

### Study limitations

The main limitation of this systematic review is the low number and quality of the studies included. The great heterogeneity of the HRV recording and analysis methods used, as well as the great heterogeneity of the population of each study prevented us to perform a meta-analysis. Other limitations are the low number of patients in each study and the fact that all of them were unicentric. Thus, although it is possible to affirm that a reduction in HRV fall seems to be related to sepsis mortality, it would be necessary to perform a larger, preferably multicenter study, to define the best HVR recordings and analysis methodology, as well as what parameters and cutoff points should be adopted to predict the risk of death.

## Conclusions

Several HRV parameters are reduced in nonsurviving septic patients in short-term recording. SDNN seems to be independently associated with mortality in sepsis, emerging as a useful HRV parameter to predict sepsis outcome. These findings need to be confirmed in larger well-designed studies.

## Supporting information

S1 File PRISMA checklist. (DOC)

S2 File PRISMA flow diagram. (DOC)

S1 Table Treatment for artifacts used in each study. (DOC)
