# Supplementary material for: Heart rate variability as predictor of mortality in sepsis: A systematic review
Source: PLoS One. 2018 Sep 11;13(9):e0203487. doi: 10.1371/journal.pone.0203487 (PMC6133362; doi:10.1371/journal.pone.0203487)
Supplement: S2 File — (DOC) [file pone.0203487.s002.doc]

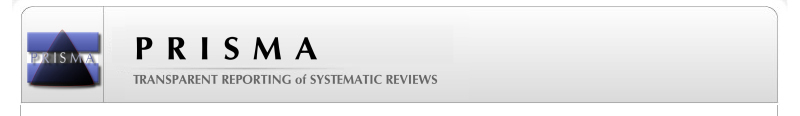
**S2 File - PRISMA Flow Diagram**

**Screening**

**Included**

**Eligibility**

**Identification**

Records identified through database searching
(n = 89 )

Additional records identified through other sources
(n = 1 )

Records after duplicates removed
(n =90)

Records screened
(n = 25 )

Records excluded
(n = 65 )

Full-text articles assessed for eligibility
(n = 9 )

Full-text articles excluded
(n =17) for difference in scope: there was no comparison between HRV in the surviving and non-surviving groups

)

Studies included in qualitative synthesis
(n = 9 )
