# Supplementary material for: Heart rate variability as predictor of mortality in sepsis: A systematic review
Source: PLoS One. 2018 Sep 11;13(9):e0203487. doi: 10.1371/journal.pone.0203487 (PMC6133362; doi:10.1371/journal.pone.0203487)
Supplement: S1 Table — (DOC) [file pone.0203487.s003.doc]

S1 Table: Newcastle-Ottawa Scale

| **Study**  **(1st author/year)** | **Artefacts** |
| --- | --- |
| Tateishi 2007 | Small number of arrhythmias were dealt with using spline interpolation, but cases in which noise and artifacts comprised more than 20% of all ECG data were excluded from the analysis. |
| Nogueira 2008 | ? |
| Chen 2008 | All artifacts or ectopic beats were deleted and replaced by interpolated beats derived from the nearest valid data before analysis. If the percentage of deletion and replacement was more than 5%, then the data of the patient were excluded from statistical analysis. |
| Papaioannou 2009 | A 128-s time series that was artifact free was chosen for off-line analysis by someone who was blind to the patient’s diagnosis. |
| Duque 2012 | ? |
| Chen 2012 | All artifacts and ectopic beats were removed, and the missing data were replaced by interpolated beats derived from the nearest valid data. If the percentage of deletion was more than 5%, then the patient was excluded from the study. |
| Brown 2013 | We identified (and excluded) ectopic and postectopic beats as well as uninterpretable segments of ECG signal (generally related to patient motion or displacement of electrodes) using a custom-written software. |
| Cedillo 2015 | ? |
| Castilho 2017 | Artifacts and irregular beats (extrasystoles, noise and missing beats) were manually deleted before analyses. There was no significant difference between the percentage of artifacts from surviving and non-surviving groups. |
